# Persistent Jaundice in Children

**Published:** 1903-09-05

**Authors:** 


### PERSISTENT JAUNDICE IN CHILDREN.

Dr. F. Parkes Weber [1] mentions a case of the above which recently came under his observation. The patient, a girl 18 years of age, of Jewish extraction, is described as having been a well-grown, fairly plump girl, with persistent slight jaundice. This jaundice was first noticed when she was 14 years old, and since it appeared she had had a tendency to bleeding from the nose and gums. No enlargement of liver or spleen could be felt. The fæces were usually well coloured, but sometimes were pale, and the urine contained urobilin. The jaundice varied in degree from time to time, but was not influenced by any treatment which was tried. The case, Dr. Weber thinks, resembles those described by French authors as simple cholæmia, though, unlike some of this class, the jaundice was not congenital and apparently did not exist in any other members of the family to which the patient belonged. The pathogeny of such cases is not known, but it seems that the jaundice may persist without leading to grave organic changes, except that the liver or spleen may become temporarily or permanently enlarged. Occasionally there are exacerbations of the jaundice, sometimes associated with pyrexia.

Dr. Weber mentions other somewhat similar cases in most of which, however, the jaundice was first noticed shortly after birth, and there was a characteristic family history of the condition. In discussing the pathology of these cases Dr. Weber places them under three headings as follows: (1) biliary cirrhosis with chronic jaundice (Hanot's disease); (2) congenital biliary cirrhosis with obliteration of bile ducts; and (3) simple persistent and congenital persistent jaundice without evidence of cirrhosis. The case above described Dr. Weber considers to be an instance of the last-named condition. In Hanot's disease the surface of the liver may be granular, but has seldom a typical "hob-nail" appearance. The cirrhotic process is generally fairly uniform; there is an increase of bile canaliculi and granules of inspissated bile-pigment are seen in the small bile ducts and liver cells. The condition is mainly one of childhood, but it may commence in later years. Sometimes several cases may occur in one family. When the disease begins at an early age it may lead to stunting of the child's growth and infantilism. It is usually associated with a tendency to bleeding from the nose, gums, etc., but articular swellings do not occur as they do in hæmophilia. The typical form of the disease is a hypertrophic cirrhosis with chronic jaundice, though the degree of jaundice may vary from time to time or even disappear for awhile. The spleen is generally much enlarged, and the splenic tumour may form one of the most striking clinical features of the case. In the class of case known as congenital biliary cirrhosis with obliteration of the bile ducts, in most instances the lumen of some portion of the bile ducts is found completely obliterated, but sometimes no evident obstruction can be found. The exact site of the obliteration, when discoverable, varies indefinitely. Many of the cases which have been described are probably instances of biliary cirrhosis due to a non-suppurative cholangitis, just as typical cases of Hanot's disease are. The peculiarity consists in the early onset of the disease. In the third class of case, which he terms simple persistent and congenital persistent jaundice without evidence of cirrhosis, Dr. Weber considers it probable that the clinical features can be explained by occlusion of one or more of the smaller hepatic ducts by a process of obliterative cholangitis. The bile-pigment from a small portion of liver substance would thus be absorbed into the circulation instead of passing into the bile ducts. This would account for the persistent moderate jaundice, in spite of the fæces being well coloured.

[1] Edinburgh Medical Journal, August.

